# Factors That Influence Use of a Patient Portal by Health Professionals

**DOI:** 10.3390/ijerph18041877

**Published:** 2021-02-15

**Authors:** Anna Janssen, Melanie Keep, Hiran Selvadurai, Andrea Kench, Sharon Hunt, Sharon Simonds, Tracey Marshall, Lucy Hatton, Luciano Dalla-Pozza, Cheryl McCullagh, Tim Shaw

**Affiliations:** 1 Research in Implementation Science and eHealth Group (RISe), Faculty of Medicine and Health, The University of Sydney, Sydney 2006, Australia; tim.shaw@sydney.edu.au; 2 Faculty of Medicine and Health, Sydney School of Health Sciences, The University of Sydney, Sydney 2006, Australia; melanie.keep@sydney.edu.au; 3 Department of Respiratory Medicine, The Children’s Hospital at Westmead, Westmead 2145, Australia; hiran.selvadurai@health.nsw.gov.au (H.S.); sharon.hunt@health.nsw.gov.au (S.H.); sharon.simonds@health.nsw.gov.au (S.S.); tracey.marshall@health.nsw.gov.au (T.M.); lucy.hatton@health.nsw.gov.au (L.H.); 4 Department of Nutrition and Dietetics, The Children’s Hospital at Westmead, Westmead 2145, Australia; andrea.kench@health.nsw.gov.au; 5 Cancer Centre for Children, The Children’s Hospital at Westmead, Westmead 2145, Australia; luciano.dallapozza@health.nsw.gov.au; 6 The Children’s Hospital at Westmead, Sydney, Westmead 2145, Australia; cheryl.mccullagh@health.nsw.gov.au; 7 Digital Health CRC, Sydney 2006, Australia

**Keywords:** digital health, patient portals, paediatric care, implementation

## Abstract

Patient portals are websites or apps that provide patients with tools to manage healthcare appointments, access their health records, and communicate with clinicians. Patient portals have been demonstrated to be beneficial for improving communication between patients/carers and their healthcare team in a range of health settings. However, there is limited research on the barriers and enablers for implementing patient portals from the perspective of health professionals and healthcare teams, particularly in a paediatric setting. This study aimed to understand healthcare teams’ experiences of using a patient portal and, using the Unified Theory of Acceptance and Use of Technology (UTAUT) framework, explore the barriers and enablers to ongoing use. Participants were 11 health professionals participating in the pilot of a patient portal for patients/carers in paediatric care. Data were collected using semi-structured interviews. Analysis of the interview data identified nine themes about implementing a patient portal in paediatric care, all of which aligned with the four constructs of the UTAUT. This study identified that barriers and enablers of the uptake of a patient portal by health professionals in a paediatric context aligned with the UTAUT framework. Value for the patient, improved workflow, and adequate technical and implementation support were highlighted by participants.

## 1. Introduction

Patient portals are internet-based, secure digital platforms integrated with Electronic Health Records (EHRs) that provide patients and/or their carers with access to a range of functionality. Owned by health services, patient portals can be websites or mobile applications (apps), and include text-based communication with healthcare providers, access to personal health information held by the healthcare provider (e.g., test results), educational resources and medication management [1]. In the paediatric setting, patient portals can provide families with greater transparency, monitoring, and collaboration with health professionals around the care and management of the patient’s health [2].

Patient portals have been consistently well received by parents of children with chronic conditions in the United States [3]. Interviews with parents who have used patient portals identified greater access to communication, reassurance, sense of control, and convenience as key benefits of patient portals [4]. Parents who communicated with clinicians via the portal (e.g., through email or SMS) indicated the communication was useful and timely [5]. Patient portals have also been shown to improve outcomes for patients through increased insight into the care process, resulting in a sense of empowerment [6].

For health professionals, patient portals have both increased their work (patients communicating more frequently because communication is easier, integrating a new system into their work) and improved efficiency (responding via text is more efficient than phone calls, and clinicians indicate greater preparedness for face-to-face clinic visits) [7]. This research, however, focuses on patient–practitioner relationships among adult patients. In the paediatric setting, this relationship is influenced by the presence of a parent or carer who would interact with the health professional on behalf of the patient [8]. To date, there is limited understanding of factors affecting clinicians’ acceptance of patient portals for use with paediatric patients with chronic conditions and their families.

Health professionals are a key source of information for patients/carers and have a strong influence on attitudes towards adoption of new health technologies by these individuals [9]. Further, the balance between the perceived impact of direct patient communication on staff work patterns and patient outcomes may affect staff decisions to promote and endorse apps to patients—a key strategy for successful uptake of apps [10]. Combined, this highlights the need for research into the experiences of health professionals who have used patient portals within a paediatric setting.

A number of theoretical frameworks exist to help contextualise the factors that influence uptake of technologies by end users [11,12]. The Unified Theory of Acceptance and Use of Technology (UTAUT) [13] is an overarching framework of technology acceptance bringing together core concepts from eight established and highly regarded technology acceptance models and frameworks. This theoretical model describes four predictors of behavioural intention: performance expectancy (individuals believe that the use of a technology will be beneficial), effort expectancy (expected ease of use), social influence (expected attitude of significant others toward using the technology), and facilitating conditions (organizational or technical resources and pre-conditions to technology use).

In the context of digital health, the UTAUT has been used to predict factors influencing patients’ intention to use telemedicine [14] and mHealth services [15]. Further, the UTAUT has also been used to predict health professionals’ intention to adopt electronic medical records (EMRs) [16,17] and electronic prescribing systems [18]. However, intention-to-adopt is necessary but not sufficient for actually adopting the tool. Research is needed to explore how perceived barriers and enablers to intention-to-adopt relates to perceived barriers and enablers during actual implementation [19].

In the context of patient portals, one review used the UTAUT to categorise findings on the intended uptake of a patient portal by older adults [20]. Beyond this, there is limited application of the framework for evaluating patient portals, particularly regarding the barriers and enablers influencing health professional uptake of patient portals. The current literature has also suggested that further research explore the UTAUT’s applicability for understanding digital technology uptake among different user groups and in different organisational contexts [13]. Context-sensitive approaches have been advocated as enable better understanding of how technology adoption models and frameworks can be applied in specific instances [17].

The aim of this study was to explore health professionals’ experiences of using a patient portal to communicate with families of paediatric patients with chronic conditions (including perceived impact on patient care). A secondary aim was to understand the perceived barriers and enablers to acceptance of patient portals by health professionals.

## 2. Materials and Methods

### 2.1. Study Design

**Figure 1:**
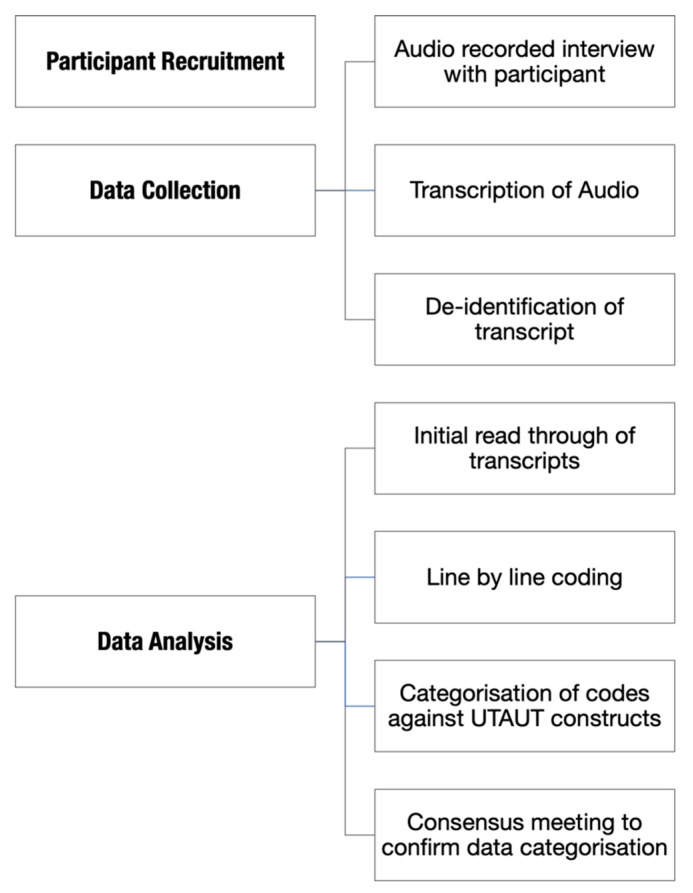
Process diagram showing how the study data was collected and analysed. UTAUT: Unified Theory of Acceptance and Use of Technology.

Thematic Content Analysis was used to analyse data collected from semi-structured interviews. The tenets of the UTAUT [13] were then deductively applied. The UTAUT was designed to be used in quantitative research to develop questionnaires based on key constructs. However, researchers have adapted the model outside this context. This has included applying the UTAUT to characterise factors in the literature that enable adoption of EMRs [21], to deductively analyse qualitative interview data [22,23] and to design semi-structured interview guides based on UTAUT constructs. Refer to Figure 1 to see a process diagram showing how the study data was collected and analysed

### 2.2. Study Setting and the Patient Portal

The study was undertaken at a tertiary children’s hospital in a metropolitan setting. In May 2017, the study site introduced a patient- and family-facing patient portal. Its initial functions included the ability to synchronize appointments with the native phone calendar, links to information about the appointment and a telehealth app; appointment rescheduling; messaging services; and automated storage of messages exchanged through the app in the patients’ EHR (held at the hospital) and in the family’s phone.

The patient portal underwent a 12-month development phase, prior to being piloted within the Cystic Fibrosis, Chronic Asthma and Cardiac Services teams. Prior to the patient portal launching, an education program was provided to patients and families. They were advised of how messages in the app would be monitored by staff. This included information that messages would be reviewed at least daily in working hours but that any emergency needs would require a phone call to healthcare providers.

During the pilot period technical support for the patient portal was provided by the vendor, not the hospital’s internal Information Technology team. Patients and families retained access to all the existing communication pathways in the hospital including email and phone calls.

### 2.3. Participants

Health professionals and administrative staff from the teams that piloted the patient portal were eligible to participate in the study. Purposive sampling was used to ensure participants had engaged with the pilot, and recruitment continued until data saturation was reached.

A total of 11 participants consented to participate in the post-launch interviews across the three participating healthcare teams. Participants were from a range of disciplines including nursing (*n =* 9), dietetics (*n* = 1), and physiotherapy (*n* = 1). Physicians did not participate in the pilot. Interviewees were from the cystic fibrosis clinic (*n* = 9), chronic asthma clinic (*n* = 2) and the heart centre (*n* = 1). All participants reported regular use of the app, though the majority of interviewees reported only using a subset of the patient portal functions (primarily the messaging function).

### 2.4. Procedures

Interviews were conducted with health professionals across the three participating clinics three months post-launch of the patient portal. Potential participants for the post- launch interviews were identified by members of the Advisory Committee that oversaw the initial pilot of the portal. A member of the research team then followed up with all eligible participants via email and organised a time to conduct a pre-launch interview.

Interviews were conducted face-to-face or via phone (30–60 min), and were audio recorded. Participants were asked to share their experiences of using the patient portal, including issues they experienced, factors which influenced their use, any feedback from patients and how the implementation experience could be improved. Audio recordings were transcribed by a commercial transcription service, prior to de-identification by one member of the research team. Once de-identified, transcripts were thematically analysed.

### 2.5. Data Analysis

An inductive analysis of the data analysis was conducted by researchers experienced with qualitative methods to identify themes emerging from the interviews. Initially transcripts were read prior to coding to obtain an overall sense of the material. Line-by-line coding of the transcripts was conducted until saturation had been reached. A deductive analysis was then used to group the codes against the four constructs of the UTAUT. Codes were then grouped by related themes and sub-themes. Iterative discussion between researchers was used to establish consensus on alignment of themes with constructs. This approach was used to understand the extent to which the UTAUT constructs comprehensively categorised data related to the implementation of a patient portal. Furthermore, the process allowed identification of any categories that may fall outside the UTAUT.

### 2.6. Ethics

The study was granted Human Research Ethics Committee approval (protocol/approval number: LNR/17/SCHN/86).

## 3. Results

According to the UTAUT [14], intention to use a tool depends on (1) performance expectancy, i.e., individuals’ perceptions of the way in which the digital technology will improve their job performance; (2) effort expectancy; (3) social influence; and (4) facilitating conditions. The results that emerged from analysis of the qualitative data are categorised in the subsequent section under these four key constructs. The categories that emerged from the analysis were:

- Experience Design (how intended functionality influenced use of the patient portal);
- Technical Reliability (unintended functionality, e.g., technical bugs);
- Health professional workflow (how the portal integrates with health professionals’ current ways of working);
- Improved efficiency for patients/carers;
- Patient Feedback;
- Technical Support;
- Integration with systems;
- Implementation Support.

Exemplar quotes of how the themes that emerged from the analysis aligned with the UTAUT constructs is presented in Table 1.

### 3.1. Performance Expectancy

Two themes emerged that related to performance expectancy: Experience Design and Technical Reliability. Experience Design described how the vendor’s intended functionality of the patient portal influenced interviewee’s ability to perform their job. Technical Reliability described how unintended functionality of the patient portal, such as bugs and errors in the technology, influenced participants’ perceptions of their job performance.

#### 3.1.1. Experience Design

The majority of participants reported positive feedback on the overall design of the patient portal, indicating the design was intuitive and made the app easy to use.

Some interviewees felt that the design of the messaging functionality, particularly the inclusion of all team members in all correspondence, enabled individual team members to improve their performance, and provide care advice with appropriate contextual information. One interviewee also stated that the app design aligned with their expectations, and would likely improve communications between patients/carers and healthcare teams.

There were also, however, design features that were considered barriers to uptake by interviewees. The absence of read receipts for messages, and poor or inconsistent notification of new messages were highlighted as challenges. For example, the absence of notifications when a new message was received through the patient portal was problematic, as messages from patients/carers could get lost or not be actioned promptly. The patient portal was also designed so that once a member of the healthcare team responded to a message future messages were sent to that team member, even if it was not directed to that person/role. A number of interviewees discussed this design choice as a major barrier to their use of the patient portal. Finally, a small number of participants reported feeling both encouraged by the potential of the app, and simultaneously constrained by its limitations. A specific example was being unable to attach documents to messages sent via the patient portal, a key component of participants’ roles. Most interviewees indicated they thought this functionality would be in future iterations of the patient portal, but wanted to be able to use it now.

#### 3.1.2. Technical Reliability

A number of interviewees cited technical reliability (ongoing glitches and bugs) as one of the only negatives of the user experience with the patient portal. Interviewees were pragmatic about the inevitability of technical glitches during a pilot period. Overall, the reliability issues were less of a concern to the health professionals themselves, but they perceived them as being a factor that would limit patient/carer uptake of the portal, and make it challenging to promote uptake in future even when the glitches were resolved.

### 3.2. Effort Expectancy

Two themes emerged relating to effort expectancy: Health Professional workflow and Improved efficiency for patients/carers. Health professional workflow describes the impact interviewees perceived the patient portal had on their ways of working, and the efficiency of processes. For participants, effort expectancy also related to the potential effort required by their patients/families. All interviewees indicated that they continued to use the portal because they perceived improved efficiency for patients/families, i.e., the patient portal enabled streamlined, flexible, and documented communication between health professionals and patients/families.

#### 3.2.1. Health Professional Workflow

Interviewees placed considerable emphasis on integrating the app with the existing infrastructure, so that additional effort required for health professionals to use the portal would be minimised.

A number of interviewees reported perceived reduced workload for managing certain types of correspondence as a result of using the patient portal. In particular, many patients used the portal for non-urgent issues, thereby reducing the number of phone calls, emails and the paging system communications.

Some interviewees also discussed the need to modify existing workflows to incorporate the demands generated by the patient portal. An example of this was staff scheduling time in the day to respond to messages, particularly as these messages were not accessible on smart phones or for remote access, so it was not possible to respond outside the hospital. For staff in multidisciplinary care teams, interviewees shared how team communication and adapting team processes to incorporate the app was a facilitator of an effective new way of working.

There was also some communication within team regarding coordination of communication and other types of feedback. One interviewee noted that it was necessary to ensure that patients/carers were aware that messages in the portal could be read by the whole team, but this was an observation rather than a barrier to or an enabler of use.

#### 3.2.2. Improved Efficiency for Patients/Carers

Interviewees reported a range of benefits for carers/patients, many highlighting improved communication from families, and more flexible options for families to ask questions and receive feedback. Participants frequently described the app as improving efficiency for the patients/families. In particular, interviewees noted that simple communication, such as checking up on test results or confirming what was needed for an appointment, was more efficient via the app. One interviewee noted that carers/patients saw value from the app as it captured longitudinal data for patients and recorded it in the electronic health record.

### 3.3. Social Influence

Feedback from patients emerged as the key theme within social influence.

#### Patient Feedback

Interviewees discussed the patient feedback extensively. Participants reported that negative feedback related to the technical reliability of the app and some concerns about the security of the patient portal. Some interviewees indicated concern that this could be a barrier for future uptake by patients/carers.

A small number of interviewees raised concerns about how the current functionality of the portal may not be equally valuable to all patients/families. These interviewees noted that it was important to ensure patients/families with different needs were made aware of the potential added value from the portal over existing systems to enable widespread uptake.

### 3.4. Facilitating Conditions

Feedback Three themes emerged that were related to facilitating conditions. Participants reported availability of technical support to introduce and maintain long term use of the patient portal, integration with systems both technical and organisational systems within the hospital, and implementation support as key factors influencing their continued use of the patient portal.

#### 3.4.1. Technical Support

The extent to which technical support was provided during the launch of the patient portal was discussed extensively during the interviews. In particular, participants highlighted having good support from the vendor during the launch of the app. This included helping families install the app, and answering patient/carer enquiries about this process. In addition, interviewees appreciated being provided with general feedback on the app, such uptake data, by the vendor.

Some participants reported concern about the sustainability of the high level of support they had been provided to date by the vendor, as technical support transitioned from the vendor to internal ICT services. Some interviewees reported a need for more training/resourcing for ICT support staff on using the portal for the transition to be successful.

By far the biggest concern interviewees raised regarding technical support was a lack of communication from the vendor about when the patient portal was not working. For example, hospital staff were not informed when the patient portal was intentionally taken offline for upgrades, or when there was an error with the messages patients received.

#### 3.4.2. Integration with Systems

Interviewees identified the extent to which the patient portal was integrated within the existing infrastructure and systems in the organisation as an enabler of uptake. Participants reported mixed responses to the system integration of the patient portal.

Interviewees also were very positive with the way the patient portal was integrated into their patient management software. This was seen as beneficial both for workflow, and for improving the quality of data captured in hospital systems, and enabling easier access this data when needed. One interviewee felt that it was quick and easy to access the portal wherever they were in the hospital.

Other interviewees felt that the patient portal was restrictive, and was only easily accessible from their desks, in contrast to emails or phone calls that could be taken from other places. This variation seemed to reflect that variability in how different specialties and professions carried out their duties.

#### 3.4.3. Implementation Support

Despite participants reporting mixed feedback about the level of support provided during implementation, they agreed that support was an important facilitating condition. Some interviewees noted good technical support in the clinics to launch the patient portal, and felt the organisation itself supported them well.

Several interviewees noted that there was limited support for implementing the patient portal beyond the first few weeks. These participants often reported reduced or slower rate of uptake from their patients due to this reduced support.

Finally, a minority of interviewees discussed the workload burden of implementing the patient portal in the clinic. These interviewees were often the ones who also felt sub-optimally supported by technical teams during the launch. Interviewees in this group discussed how busy the clinic was, and how their priority had to be on supporting the patients with their health needs, not installing the patient portal on the patient’s devices.

## 4. Discussion

This study aimed to understand health professionals’ experiences of using a patient portal for paediatric patients with chronic conditions and their families, and to apply the UTAUT to analysing semi-structured interview data in order to frame the factors that facilitated staff adoption of a patient portal. Overall, health professionals and administrative staff reported positive experiences with using the patient portal for coordinating appointments and engaging in text-based communication with parents of children with chronic conditions. The factors that affected their use aligned with the tenets of the UTAUT framework.

Findings from this study suggest that the construct of social influence is particularly important in adoption of patient portals by health professionals. Interestingly, the key social feedback was from patients. This finding deviates from the existing literature on health professional adoption of which has suggested that social influence is a not as influential as other constructs, particularly performance expectation, in determining technology adoption [18]. It is unclear why this is the case, though it is possible that since one of the core functions of a patient portal is to facilitate communication between healthcare teams and patients/carers, social (patient) feedback is emphasised in our setting. Alternatively, it may simply be a consequence of evaluating the patient portal during a pilot phase soon after implementation. The literature indicates that the social influence construct is more influential when a technology is new and the influence decreases over time [13]. Findings also emphasise the importance of technical and implementation support during the implementation phase of health technology. This finding aligns with literature showing that the process by which digital health are implemented influences uptake, and if not appropriately supported can have a negative impact on staff opinion [3].

Furthermore, this study illustrates the versatility of the UTAUT model in several ways. Firstly, it demonstrates that potential utility of the model for framing qualitative data around adoption of health technologies by health professionals. This is important because implementations of health technologies are often with sample sizes too small to capture in traditional UTAUT questionnaires, but this is often the context in which these technologies are implemented in healthcare teams. Secondly, findings from this study illustrates the importance of facilitating conditions such as technical support in sustained adoption of digital health by health professionals in the paediatric context. The identification of contextual factors that may influence adoption is a recognized strength of the UTAUT, particularly for helping understand different approaches for implementing health technology [21].

This study also demonstrated that healthcare teams were supportive about having a new tool for communicating with patients and families. Results from this study suggest that, in the context of general enthusiasm for the patient portal app, health workers had some concerns about how communication via the portal might be facilitated in the existing multidisciplinary team structure, the technical reliability of the portal (acknowledging that they are in a pilot phase), and having adequate, ongoing support. Despite these concerns, healthcare workers felt that the app would be a valuable tool for patients and families, and, as a result, they were open to exploring how it could be integrated with the workflow of all health teams. This finding is consistent with literature showing that perceived improvements to the quality of patient-clinician interactions can be an enabler of uptake [24].

A limitation of this study is its focus on an area of clinical practice that is experiencing rapid change: digital health usage in a tertiary setting. As such, it may be challenging to generalise findings across the health sector. Findings report on patient portal usage at a specific moment in time and may not reflect current usage in the organisation or usage at other organisations. However, this limitation commonly occurs in research relating to implementation of technology in health services. Future researchers may wish to use this methodology at different tertiary organisations to understand whether similar factors influence uptake of patient portals across the health sector. Researchers may wish to undertake a study of patient portal adoption by health professionals using a quantitative questionnaire that embodies the UTAUT constructs, as in this study the UTAUT was applied deductively to qualitative data. Finally, researchers may wish to undertake ethnographic research within the health system over an extended period of time, such as several years, to understand how patient portal use is integrated and sustained over time.

## 5. Conclusions

This study identified that barriers and enablers of the uptake of a patient portal by health professionals in a paediatric context aligned with the UTAUT framework. Value for the patient, improved workflow, and adequate technical and implementation support were highlighted by participants. The current research also demonstrates the applicability of the UTAUT for understanding health professional uptake of patient portals, extending current understanding of this framework. Future research could explore patient perspectives, including the extent to which patient portals are used in the long term and the impact they have on patients’ experiences of care and self-management using the UTAUT framework. Another key conclusion is that when implementing new digital health solutions it is important to get constant feedback from end-users and be able to iterate on solution based on this feedback. Digital health implementations should include mechanisms to capture this feedback and iterate on the solution to rapidly improve uptake by the health workforce and ensure it is sustained.

The study contributes two pieces of new knowledge to the literature. Firstly, it is the first study to use the UTAUT to deductively analyse qualitative data on health professional adoption of patient portals, though the framework has been applied to other forms of health technologies [17]. Secondly, this study identified factors that influenced adoption of a patient portal with features including builds on previous (limited) research on factors influencing health professional adoption of patient portals in paediatric settings.

## Figures and Tables

**Table 1:** Outline of how UTAUT (Unified Theory of Acceptance and Use of Technology) constructs aligned with sub-categories of data, aligned with exemplar quotes.

UTAUT Construct	Data Categories	Exemplar Quotes
Performance Expectancy	Experience Design (how intended functionality influenced use of the patient portal)	“I think it looks good on the phone. I think you can see how many messages you’ve got, just like you can if you’ve got emails or something.”
		“I like the way that you can skip group conversations, so I can read in context… I can read the nursing part, the physio component, and then I can read where my part fits in. I think the families also seem to really like it…”
	Technical Reliability (unintended functionality, e.g., technical bugs)	“I believe that if it’s working the way it’s meant to be working, they can reschedule appointments, or they can request to reschedule appointments, it pops up into their calendars, it’s meant to allow them to have longer or maybe better communication with us.” “We’ll send that initial message out to them, and then we don’t get confirmation there that they’ve received it. So, initially we weren’t sure whether that was because there was a glitch happening at the time, or whether they just hadn’t got it.” “It’s difficult if it’s a one-on-one communication that only comes back to that person, but it needs to be addressed or someone else needs to know about it, needs to be actioned, so that was one of the limitations.” “I think the frustration at this point is that the communication’s getting there and I can see it’s got potential, but there’s so much more that the app can do. And to keep people engaged, I think we need to start rolling out some of those other features. We can’t roll those features out until we fix the glitches, so I feel like it’s slightly losing momentum a little bit.” “So, the challenges would be, what we’ve all had, is with the app crashing. So, we got a lot of people signed up, and then we had quite few consecutive glitches, and we really lost quite a bit of their confidence in using the app.” “There’s been issues where the actual app has crashed…until all of this is running smoothly, I don’t think we will completely get people on board.”
Effort Expectancy	Health professional workflow (how the portal integrates with health professionals’ current ways of working)	“I really like the fact that it’s built into [EMR] and that whatever is typed automatically goes into the notes, so there’s no copying and pasting you need to do from emails or from text messages; that becomes an instant part of the clinical progress notes. That’s really important for workflow and for absolute transparency to when things are happening in a live situation.” “I’ve had less pages for sure. I’m sure I’ve had less pages about little things, like can I have a script? What was my result? … They know if they message me in the app and it’s, “My child is sick”, that I will probably ring them as opposed to reply in the app because I will want more information about that. It’s worked really well for me like that.” “…to come back to our office and sit down, and then look at that [the patient portal]. You know, often we have lots of interruptions, so to spend the time, and just make the part of you do it, I think that’s the challenge, I think that’s what we have to do.” “That’s not something that’s currently built into my day at all [checking messages], and I would have to say that, really, I’ve been very reliant on somebody else flagging that there’s a message for me … And mainly because our days can look very different; that’s a big concern, because there might be days where I won’t actually sit in front of a computer until midday. And yes, I do have a little concern with that.” “We kind of look after half the patients each. So we’re able to manage the communication by just us using it. The dietician and physio have used it very occasionally, but they tend to not use it too much, because they’re not in <the EMR> as often as us. So if they send a message, and a message gets sent back, and it’s for us, it’s going to sit in their inbox until they notice it.” “part of the issue around this is that we are all doubling up on … I don’t know, I’m assuming it’s one of the issues that they’re concerned that clinicians are doubling up on communication and I believe the app is meant to be how we communicate. That’s what the hospital wants, I think.” “The families need to remember that what they’re saying can be read by everyone. So, I think one family made a comment to one of the nurses the other day along the lines of, “This is great, you’re the best team.” We’ve had type of thing. But then people are sending kids, eventually we’ll be able to … It’s just they’ve got to be careful that you don’t offend other health professionals. And it will go both ways.”
	Improved efficiency for patients/carers	“I think because it’s easy for them to send me a quick message they might be messaging more readily when they might have gone, “Oh, it’s too hard to try and page her and wait for her to ring back” and all of those sorts of things.” “I like that it has made it easier for me to communicate simple things to families rather than have to get into phone calls. It’s easier for families to communicate simple things to me. Definitely more families are using the app than used to email me.” “They can understand and appreciate how it goes into their child’s medical record and they also like that they can go back and read what they’ve sent me, and what my response has been, and that stays on their app, and they know that stays there for me as well. That’s been really good.”
Social Influence	Patient Feedback	“I think that the patient group in general are really impressed with it, and like it as an option, and understand that it’s in its basic phases, and are really quite happy with it.” “I think one of the families was concerned about with information that’s going back and forward, and the security around that information, and obviously, it’s very secure, but they just had an issue with that. That’s the only reason behind not accepting, that I know of.” “Definitely families need to see more benefit from it. There needs to be more for them, I think for the complex kids. I think you’ll find … when this goes live throughout the hospital, the majority of families that will be utilising this will be the frequent fliers, and it’s the frequent attenders that need to see a benefit. So, it probably needs to be solved to them. That probably needs a lot more support to sell it to them, support them, continue to get them to see the benefit of it, and also the support for us as well.”
Facilitating Conditions	Technical Support	“They’ve been good. We’ve used [the vendor] as our first port of call and [the vendor] would tell us how to action it.” “[The Vendor] does feed back to us, they were sending through graphs and lists after clinic with who was on it, and then he was following up with who had actually activated it properly… Haven’t had that for a few weeks, but in the initial phase, he was giving stats of what percentage we had taken, things like that.” “I think that they’ve been quite supportive. [The vendor] is the main person who we deal with, and they’ve has been really supportive.” “[The Vendor’s] been very helpful, but our IT department, there seems to be a bit of a gap between [their] knowledge and understanding, and what they would know to do, and what they actually did know to do, because a few times they were just like, no, we don’t know.” “Sometimes or when we would ring a patient, or they would ring us, they would say, ‘We sent you a message earlier today about dah-dah-dah-dah-dah-dah.’ Then they’d keep talking about and we’d go, ‘Oh, we didn’t get a message earlier today from you.’ That kind of thing.” “If I am not sure that something is working, I’m not going to use something. Especially if I have to send information about an appointment or about a change in medication or something.” “I guess, as would be expected, when you’re trialling something, there have certainly been some teething issues…There’s also been a fair bit of time where it’s gone down. The concern for me around that is that families at the other end trying to use it, and it’s not working properly. So they get frustrated and revert back to the old means of communicating with us.”
	Integration with systems	“On the ward they’ve got the computer’s on wheels, and in a lot of the rooms there’s the fixed computers in the patient care areas, so it’s pretty easy to access anywhere within the hospital.” “I can jump onto [EMR] at any time, so if I’m on night duty in another job, I can log into power chart and find out what the follow up was from that outcome that I knew was being looked into on the Tuesday, or whatever, so it does keep me in the loop, and it’s an easy way to just be able to jump onto power chart any time. You know, midnight, three in the morning, it’s not restrictive like it would be with a telephone. So, it’s good for that way of sharing information around and having it accessible and visible there to easily go through and track the conversation.” “I think the thing for us is that… we have email on our phones, so we’re out and about, we’re not always at our desk and you have to log on. So I think that the beauty for us is that when we have patients emailing us, we have the opportunity on our phone to actually look at it, and respond almost immediately. Whereas we don’t have the opportunity to log on to Powerchart on our phones.”
	Implementation Support	“There’s been a lot of good ideas, and plans, and projects in this hospital, but I think there was the appreciation and the realisation that staff just can’t keep taking on extra thing and getting them up and going for people. If you want to get something up and going properly, you need to put in the resources to do it and they did do that, which was excellent. I’m glad they had the foresight to do that, because it’s really good and it would’ve been another thing that fell down if it was allowed to happen naturally, it wouldn’t have…” “Initially it was all very positive. We got lots of information. There was [vendor] staff that were there at the clinic to help with the initial trial in chest and asthma clinic.” “In order to get more uptake for more patients, you actually need more people on the ground who are just doing that… to expect clinicians to do that as well as their clinical work is unrealistic, especially if you really want to roll something out. We’re very happy to mention it, here’s the information on it, but we can’t spend 10 min explaining it, helping them putting it on their phone. We’re in the middle of a clinic. We might be trying to see 10, 12 patients in a half hour, in a two-hour clinic, so we can’t do that.”

## Data Availability

Data is available upon reasonable request by contacting the corresponding author.
